# GRPR-targeting radiotheranostics for breast cancer management

**DOI:** 10.3389/fmed.2023.1250799

**Published:** 2023-11-03

**Authors:** Alice D’Onofrio, Swantje Engelbrecht, Tilman Läppchen, Axel Rominger, Eleni Gourni

**Affiliations:** Department of Nuclear Medicine, Inselspital, Bern University Hospital, University of Bern, Bern, Switzerland

**Keywords:** gastrin-releasing peptide receptor (GRPR), Bombesin, breast cancer, molecular imaging, peptide receptor radionuclide therapy (PRRT)

## Abstract

Breast Cancer (BC) is the most common cancer worldwide and, despite the advancements made toward early diagnosis and novel treatments, there is an urgent need to reduce its mortality. The Gastrin-Releasing Peptide Receptor (GRPR) is a promising target for the development of theranostic radioligands for luminal BC with positive estrogen receptor (ER) expression, because GRPR is expressed not only in primary lesions but also in lymph nodes and distant metastasis. In the last decades, several GRPR-targeting molecules have been evaluated both at preclinical and clinical level, however, most of the studies have been focused on prostate cancer (PC). Nonetheless, given the relevance of non-invasive diagnosis and potential treatment of BC through Peptide Receptor Radioligand Therapy (PRRT), this review aims at collecting the available preclinical and clinical data on GRPR-targeting radiopeptides for the imaging and therapy of BC, to better understand the current state-of-the-art and identify future perspectives and possible limitations to their clinical translation. In fact, since luminal-like tumors account for approximately 80% of all BC, many BC patients are likely to benefit from the development of GRPR-radiotheranostics.

## 1. Introduction

Breast Cancer (BC) is the most common cancer worldwide with incidence rates that have been slowly increasing since the mid-2000s by about 0.5% per year (1). Currently, more than 90% of BC have the potential to be diagnosed at an early stage and before the insurgence of metastatic spread, thus leading to successful therapeutic outcomes in approximately 80% of the cases (2). However, despite these accomplishments, there is an urgent need to reduce BC mortality as the treatment of advanced BC with distant organ metastases is challenging and with limited successful therapeutic approaches (30% 5-year Relative Survival Rate according to the American Cancer Society) (3). In the past 20 years, the heterogeneity of BC at molecular level has been extensively characterized and the information provided has been successfully used for the design of personalized therapeutic regimens with improved efficacy, that have contributed to significantly enhance the subtype-specific survival (4). The biomarkers involved in BC include immunohistochemical markers [estrogen receptor (ER), progesterone receptor (PR), human epidermal growth factor receptor-2 (HER2) and the proliferation marker protein Ki-67], genomic markers [BReast CAncer gene 1 and 2 (BRCA1, BRCA2 and Phosphatidylinositol-4,5-Bisphosphate 3-Kinase Catalytic Subunit Alpha (PIK3CA)] as well as immunomarkers [tumor-infiltrating lymphocytes and Programmed Death Ligand-1 (PD-L1)] (5).

The advancements made in the discovery of novel targeted treatments for BC have allowed to explore several biomarkers suitable also for molecular imaging, which has ultimately contributed to a better BC management through more accurate diagnoses, treatment planning and therapeutic follow-ups. Specific radiotracers for the imaging of ER, PR and HER2 receptors allow the non-invasive evaluation of biomarker expression during the course of the disease, overcoming some of the limitations associated with biopsies, namely lesion heterogeneity and technically challenging sampling. On the other hand, PET/CT imaging using the non-specific tracer 2-deoxy-2-[^18^F]fluoro-D-glucose (2-[^18^F]FDG) is the current state-of-the-art for the evaluation of the metastatic spread and has demonstrated higher efficacy for the detection of regional and distant metastasis when compared to morphological imaging. Nonetheless, this modality has some inherent limitations, such as a relatively low detection rate of bone metastases, especially in case of the sclerotic subtype, and a relatively high rate of false positive results (6). For the initial staging of BC, 2-[^18^F]FDG PET/CT has also demonstrated to be useful from clinical stage IIB, regardless of tumor phenotype and despite some limitations in the case of low proliferative tumors, low-grade tumors and for well-differentiated luminal BC (7).

Other molecular targets, such as the somatostatin receptor (SSTR), gastrin-releasing peptide receptor (GRPR), folate receptor (FR), C-X-C chemokine receptor type 4 (CXCR4), neuropeptide Y receptor Y1 (NPY1R) and vasoactive intestinal polypeptide receptor 1 (VIP-R1) have been previously evaluated for their potential for BC imaging (8, 9). Considering the recent approval for clinical use of other peptide-based radiopharmaceuticals, GRPR is a particularly promising target for the development of diagnostic and therapeutic radioligands because of its very favorable expression pattern in several tumors, including BC. Many preclinical studies on GRPR-targeting molecules have been reported in the last decades and some of these compounds are currently being evaluated in clinical settings. Most of the studies have been focused on the development of GRPR radiopeptides for prostate cancer (PC) theranostics, also because of their important role in tumors with low Prostate Specific Membrane Antigen (PSMA) expression (10–14). However, a considerable body of evidence suggest that GRPR-targeted imaging might be useful for the non-invasive disease staging and therapy evaluation in ER-positive BC patients, which might be also translated into potential treatment of BC through Peptide Receptor Radioligand Therapy (PRRT) (15, 16). Given this context, this review aims at collecting the available preclinical and clinical data on GRPR-targeting radiopeptides for the imaging and therapy of BC, focusing on the current state-of-the-art, future perspectives and possible limitations to their clinical translation.

## 2. Molecular BC subtypes and clinical management

**Figure 1:**
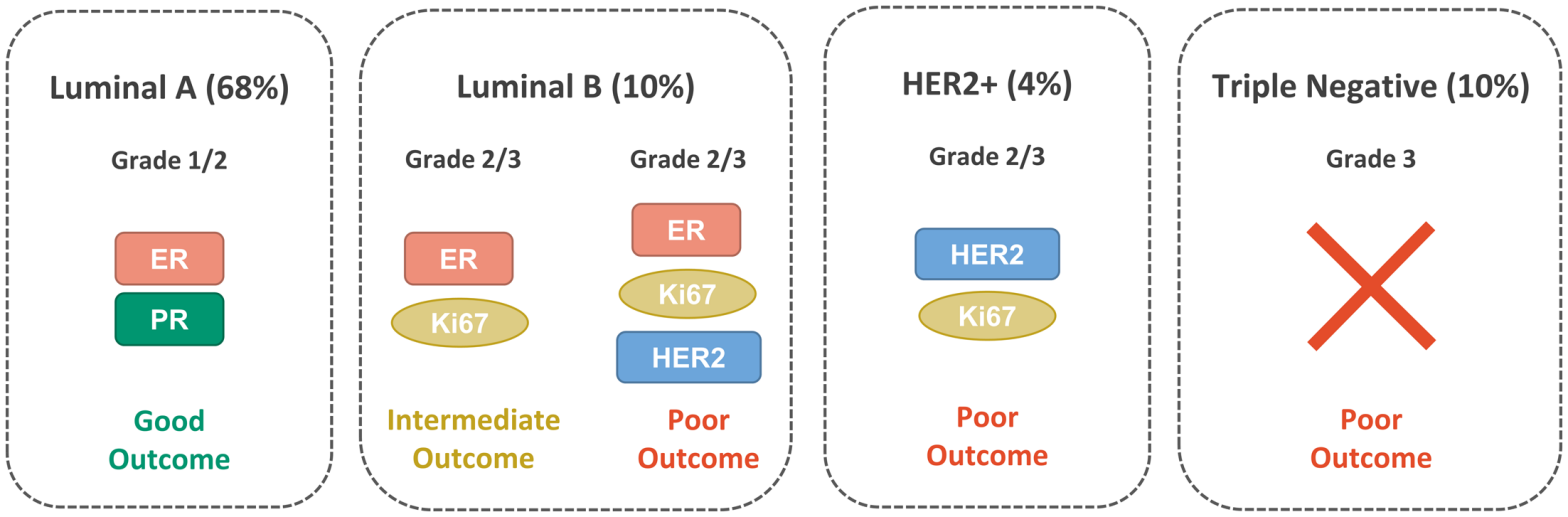
Main BC molecular subtypes: Luminal A, Luminal B, HER2-positive and TNBC with their clinical characteristics and the biomarkers expressed.

Several molecular BC subtypes have been described from an immunohistochemical perspective and based on the involvement of specific hormone and growth factor receptors: luminal A, luminal B, HER2-enriched and triple-negative (TNBC or basal-like), as shown in Figure 1. However, the classification of the subtypes has continuously evolved over the years and is still controversial (17). Luminal A expresses both ER and PR, is HER2-negative and possesses low levels of the protein Ki-67. It has the tendency to grow at a slower pace than the other subtypes and generally has a good prognosis (18, 19). Luminal B is generally ER-positive, PR-negative and can be either HER2-positive or negative. It has a fast proliferation rate, as indicated by the high levels of Ki-67 and generally have worse prognosis then luminal A. The HER2-enriched subtype has low expression of ER and related genes and is HER2-positive. Generally, it has a fast proliferation and is associated with a worse prognosis but tends to respond well to HER2-targeted therapies. TNBC is ER-, PR- and HER2-negative and is often more aggressive than either luminal A or luminal B (20–22).

BC management is complex and requires a multidisciplinary approach that includes surgery, radiation therapy and systemic therapy. However, aiming to improve the quality of life of the patients, de-escalation schemes have recently become the standard of care, with safe and effective conservative approaches and the implementation of neoadjuvant chemotherapy regimens (23). Adjuvant therapies are planned according to the different gene expression patterns of the BC subtypes and the corresponding differences of the tumors at molecular level (24). In particular, the status of specific predictive markers, such as ER or HER2, is evaluated to define the most suited systemic therapy. Adjuvant systemic treatments might include endocrine therapy for ER/PR-positive disease, anti-HER2 therapy for HER2-positive disease, chemotherapy for TNBC to reduce the risk of relapse and poly (ADP-ribose) polymerase (PARP) inhibitors for BRCA mutation carriers (25–28). For metastatic BC, standard therapy options include targeted approaches such as CDK4/CDK6 inhibitors, PI3K and PARP inhibitors and anti-PD-L1 immunotherapy, depending on tumor subtype and molecular profile (29–32). To further improve the therapeutic outcome and reduce the risk of recurrence, novel targeted treatments and their combinations with existing therapeutic regimens, are also being extensively explored (33). Nonetheless, current BC treatments have severe adverse effects and patients can easily acquire resistance to endocrine therapy, anti-HER2 therapy and chemotherapy. Moreover, the TNBC subtype, which has the lowest survival rate, lacks a standardized therapy (34).

The diagnosis and staging, in most cases, are performed through anatomical imaging techniques such as mammography, ultrasound, magnetic resonance imaging (MRI) and computed tomography (CT), followed by histological analysis to determine the biomarkers involved. However, such imaging techniques present several limitations, including the inability to provide tumor-specific biochemical information. On the other hand, biopsies are invasive and have limited capability to represent tumor heterogeneity due to single-site sampling (8). Nonetheless, since tumor invasion and metastasis are highly related to the biomarkers expression, early and accurate diagnoses are essential to enhance BC survival rates. In this regard, molecular imaging is a non-invasive technique very appealing for oncology, due to its high sensitivity and the possibility to obtain a precise and personalized therapy accompanying diagnosis aiming to a patient-specific treatment.

Molecular imaging using the widespread tracer 2-[^18^F]FDG allows the visualization of all tissues with enhanced metabolic activity and 2-[^18^F]FDG PET/CT modality is widely used to diagnose and stage several tumors, including BC, and to reveal potential biopsy sites (35). 2-[^18^F]FDG PET/CT is especially relevant for the TNBC subtype because of the lack of any specific markers and early hematogeneous spread without lymph node metastasis. In a retrospective study, the usefulness of 2-[^18^F]FDG PET/CT for the early staging of TNBC has been highlighted and unsuspected metastases were detected in 15% of patients with stage II TNBC (36). In a recent study, 2-[^18^F]FDG PET/CT have also demonstrated, when compared to contrast-enhanced CT, to be a better predictor of progression-free and disease specific survival for monitoring metastatic BC. A low concordance between the two modalities was found for response categorization, suggesting that additional investigations are needed to identify the modality granting the patients a more accurate follow-up and, consequently, a better management of the disease (37). Breast-specific gamma imaging (scintimammography) using the non-specific SPECT tracer [^99m^Tc]Tc-sestamibi, generally used for myocardial perfusion imaging, has also been successfully used to detect BC because of the tracer accumulation in malignant breast tissues (38).

For the BC subtypes that overexpress ER, molecular imaging using 16α-[^18^F]-fluoro-17β-estradiol ([^18^F]FES) has been recognized as a valuable tool to overcome clinical dilemmas, when distant metastasis cannot be safely reached for biopsies sampling or when ER heterogeneity is suspected between tumor lesions (39). In addition, since the ER expression level often changes in response to the therapies or during the progression of the disease, [^18^F]FES is a useful tool for measuring ER occupancy by means of PET (40). These favorable features have contributed to the approval of the radiopharmaceutical by the FDA in 2020, while PR-targeted imaging agents, such as [^18^F]-fluoro-furanyl-norprogesterone ([^18^F]FFNP), are currently undergoing clinical trials (41).

On the other hand, the anti-HER2 monoclonal antibody trastuzumab is part of treatment in both the adjuvant as the metastatic setting of HER2-positive BC. Nonetheless, since the therapy is expensive and has considerable side effects, monitoring the status of HER2 expression is crucial to select patients likely to benefit from the treatment. In this regard, the SPECT tracer [^111^In]In-trastuzumab and the PET tracers [^89^Zr]Zr-trastuzumab and [^64^Cu]Cu-trastuzumab are being evaluated in clinical studies to determine their added value in therapy prediction and selection of patients with metastasized BC (40, 42). Furthermore, [^177^Lu]Lu-trastuzumab has been evaluated in a preliminary clinical study carried out in 8 cancer patients with HER2-positive metastatic BC, showing the preferential localization of the tracer in tumor lesions that warrant further studies for the evaluation of its therapeutic potential (43).

## 3. GRPR expression in BC

**Figure 2:**
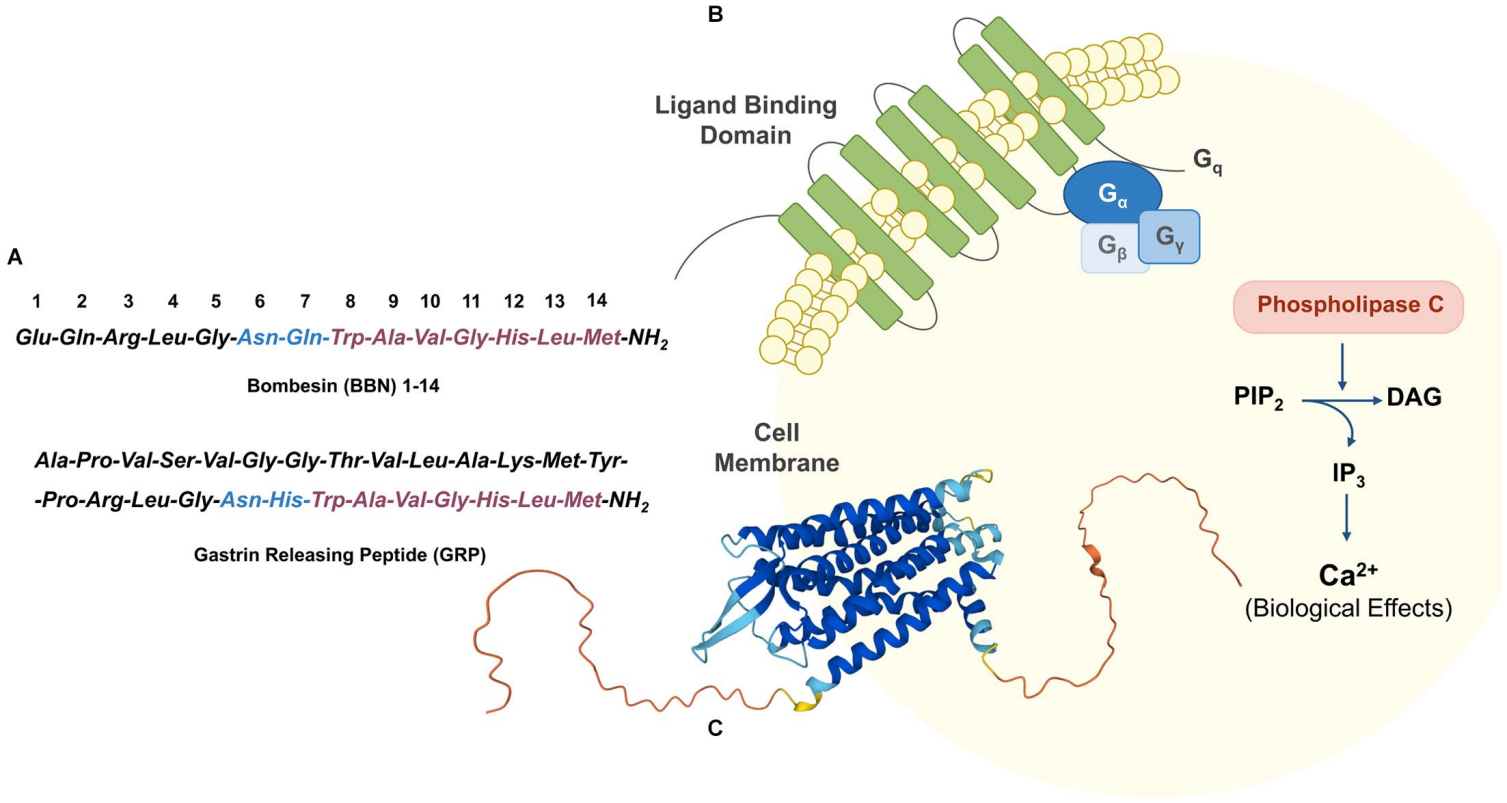
**(A)** Amino acid sequence of BBN and its mammalian counterpart GRP. **(B)** Schematic GRPR activation through phospholipase C signaling pathway. **(C)** 3D-structure of GRPR as predicted by AlphaFold (UniProt P52500) (44, 45).

GRPR, also known as BB2, belongs to the bombesin (BBN) receptor family and to the superfamily of the G-protein coupled receptors. The discovery of GRPR follows the isolation of the biologically active 14-amino acids peptide bombesin from the skin of the European fire-bellied toad *Bombina Bombina*. The corresponding mammalian counterpart, the 27-amino acids peptide GRP, which was isolated only 10 years after, shares with BBN a strongly conserved domain crucial for the biological activity (BBN (6–14)), as highlighted in Figure 2A (46, 47). GRPR is composed by seven transmembrane domains (schematic drawing in Figure 2B and the GRPR 3D-structure as predicted by AlphaFold in Figure 2C) and is activated upon the binding to an agonist, resulting into a downstream activation of the phospholipase C signaling pathway. The signal transduction is initiated by the cleavage of phosphatidylinositol 4,5-bisphosphate (PIP_2_) into diacyl glycerol (DAG) and inositol 1,4,5-trisphosphate (IP_3_), which serve as a second messenger and lead to the mobilization of intracellular Ca^2+^ ions, as shown in Figure 2B (48).

The biological effects mediated by GRPR are diverse and include the release of hormones from gastrointestinal and endocrine organs, the contraction of smooth muscles and the central regulation of temperature and circadian rhythms (49). Most importantly, GRPR activation seems to be involved in the regulation of the immune response and in the mitotic activity of human tumors (50). In normal tissues, GRPR is mostly expressed in the pancreas but lower GRPR expression is also found in the colon, breast, prostate, and central nervous system (51). Moreover, GRPR overexpression has been found in a large spectrum of human cancers including small-cell lung carcinoma, breast, stomach, colon and prostate cancer which renders it an appealing target for the development of novel peptide based radiopharmaceuticals for oncological applications (52).

**Figure 3:**
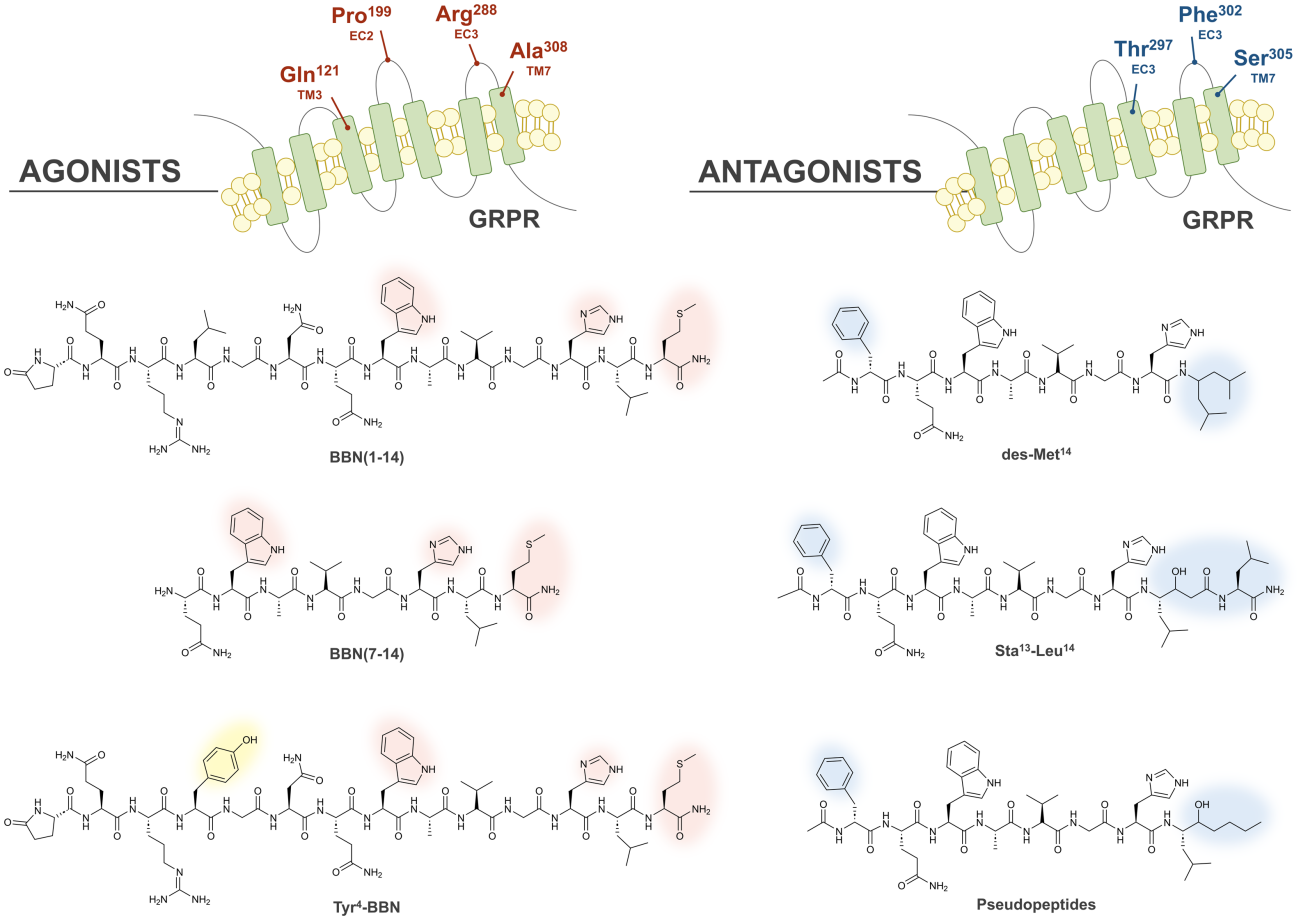
Illustration of the GRPR binding pockets of agonists and antagonists. The crucial residues forming the GRPR binding pockets of agonists and antagonists are highlighted in red and blue, respectively, at the top of the Figure. The chemical structures of the main classes of agonists and antagonists originated by different modifications to BBN are also displayed, and the amino acid essential for the agonistic or antagonistic behavior are highlighted with red and blue shadows, respectively. Substitution of Leu^4^ with Tyr allows the synthesis of radioiodinated derivatives (yellow shadow).

The role of GRPR in BC development and growth was described for the first time in 1991, after observing that the addition of BBN to four different BC cell lines induced a significant enhancement of their proliferation when compared to controls (53). Few years later, a strong positive correlation was also observed in BC tissues between high ER and GRPR expression (54). In 1999, the role of GRP as a stimulatory growth factor in human BC was further elucidated, by autoradiography studies performed with the radioiodinated bombesin analog [^125^I]I-Tyr^4^-BBN (please see the corresponding structure in Figure 3, yellow shadow), that exhibits high and specific GRPR affinity, and the universal [^125^I]I-dTyr^6^-βAla^11^-Phe^13^-Nle^14^-BBN (6–14), that binds to all four BBN receptor subtypes. High GRPR expression (62%) was verified both in primary BC and in lymph node metastasis (55).

**Figure 4:**
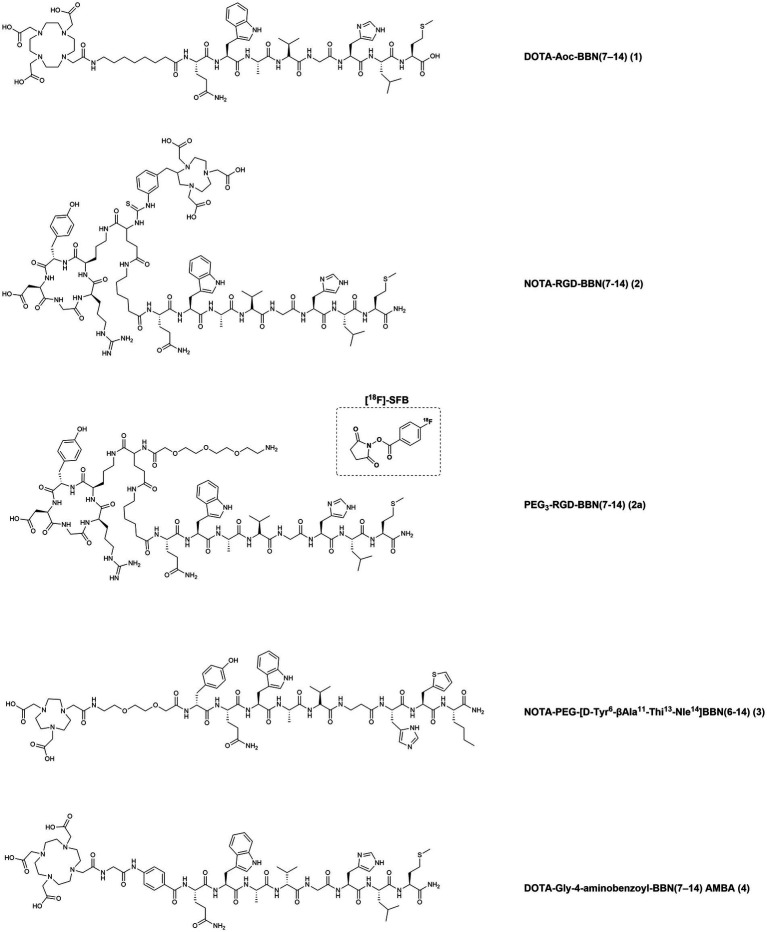
Chemical structures of the GRPR-targeting agonists 1–4, evaluated in preclinical studies upon labeling with suitable radionuclides.

To identify patients with high GRPR expression, several techniques have demonstrated to be useful such as autoradiography of frozen BC biopsies, immunohistochemistry (IHC) analysis of formalin-fixed paraffin-embedded material or messenger RNA (mRNA) and quantitative reverse transcriptase polymerase chain reaction (RT-qPCR) analysis (56). In 2015, a strong and positive correlation between the binding of the specific GRPR-based radiotracer [^111^In]In-AMBA, (AMBA: DOTA-Gly-4-aminobenzoyl-BBN (7–14) (see Figure 4, **4**)) and mRNA expression was demonstrated through *in vitro* autoradiography of clinical BC specimens. These findings were further confirmed by RT-qPCR that detected high GRPR mRNA levels in the ER-positive BC subtypes and demonstrated the high potential of this technique for the stratification of BC patient groups likely to benefit from radioligand imaging and/or therapeutic applications (57).

In a later study, GRPR overexpression was found in 76% of primary BC samples by IHC and again a strong correlation was observed between GRPR and ER overexpression, in agreement with the previous findings (high GRPR levels in 83% of ER-positive and 12% of ER-negative tumors). High GRPR expression was observed not only in the primary tumors of the BC subtypes luminal A and B (86 and 70%, respectively) but also in the 95% of the analyzed metastatic lymph nodes (58). Besides being expressed by a very high percentage of primary tumors and corresponding metastasis, GRPR also showed considerably high receptor density in the analyzed breast cancer specimens (74% of tumors analyzed expressed GRPR with a mean density of 9,819 ± 530 dpm/mg tissue). These very favorable features make GRPR particularly appealing for radioligand-based targeted imaging and therapies in ER-positive BC (59).

## 4. Development of GRPR-targeting radiopeptides

The first GRPR-based radiopharmaceuticals were peptidic analogues with agonistic profile obtained by isolation of the seven C-terminal amino acids of bombesin (BBN (7–14)), required for GRPR activation (60). Structure–activity relationship studies demonstrated that both the Trp^8^ and His^12^ residues are essential to retain bombesin-like activity, while the Met^14^ residue at the C-terminal is essential to have an agonistic behavior (61). In particular, the carbonyl group at the position 14 promotes the formation of an intramolecular H-bond essential for the activation of the receptor, in agreement with the model proposed by Coy in 1988 (62). In the transmembrane domains (TM) 6 and 7 and in the extracellular loops (EC) 1, 2, and 3 of GRPR are located several amino acids crucial for the high affinity binding of the endogenous agonist GRP (63). In particular, the substitution of four amino acids (Gln^121^, Arg^288^, Ala^308^ and Pro^199^) in the GRPR sequence resulted into a considerable decrease in the affinity for both endogenous ligands BBN and GRP, suggesting the importance of these residues in forming the agonist-binding pocket, as highlighted in red in Figure 3 (top-left) (64).

Initially, the high binding affinities of the reported agonists and their internalization into the cancer cells were expected to provide better target-to-background ratios and a prolonged pharmacological effect. Therefore, many different GRPR-based radioagonists have been developed and evaluated preclinically over the course of the years, based on the full BBN (1–14) or truncated BBN (7–14) sequence, as shown in the left side of Figure 3. Substitution of the Leu^4^ with a Tyr demonstrated to retain the biological activity and allowed to obtain radioiodinated derivatives, as shown in Figure 3.

Different clinical trials have been carried out using radiolabeled GRPR agonists, but none of them has been successfully translated into the clinical routine. In fact, prolonged exposure to radiolabeled GRPR agonists led to chronic desensitization and a fast down-regulation of the receptors (65). In addition, significant side effects were observed, including abdominal cramps and vomiting but also mitogenic properties (66–68). Therefore, the need to generate new radiolabeled GRPR analogues, which could be safely administrated to patients, led many research groups toward the development of radiolabeled GRPR antagonists. The binding of antagonists to GRPR does not trigger the activation of the receptor nor the following cascade response leading to the insurgence of side effects and has even demonstrated antiproliferative effects on several cancer models. Interestingly, radiolabeled GRPR antagonists also demonstrated high tumor accumulation *in vivo* and even superior pharmacokinetic properties, mainly due to their faster clearance from pancreas and non-target organs (69).

Many GRPR antagonists were obtained by removing the carbonyl residue at the C-terminal position and consequent disruption of the active conformation of agonists, as shown in the left side of Figure 3. This was achieved first by truncation of the terminal Met and by replacing it with C-terminal ethylamides, originating the desMet^14^ derivatives. A similar effect was obtained by introducing in the same position a pseudopeptide bond (70–72). Furthermore, several potent antagonists with improved metabolic stability were obtained by replacing the C-terminal residues Leu^13^-Met^14^ with the dipeptide Sta^13^-Leu^14^, containing the γ-amino acid statine ([3S, 4S]-4-amino-3-hydroxy-6-methylheptanoic acid) (73). Chimeric studies have highlighted that the GRPR region from the N-terminus to the end of TM2 and the regions in the EC4 and TM7 are the most involved in the interaction with antagonists. Further studies with receptor chimeras, site-directed mutagenesis and molecular modeling, also confirmed the importance of the EC4 region and in particular, for antagonists binding, the involvement of the residues Thr^297^, Phe^302^ and Ser^305^, which are highlighted in blue in the upper right part of Figure 3 (72, 74). In several GRPR antagonists the replacement of the Asn residue at the position 6 with a D-Phe led to derivatives with considerably improved potency (75).

## 5. GRPR-targeting radioligands in BC

**Figure 5:**
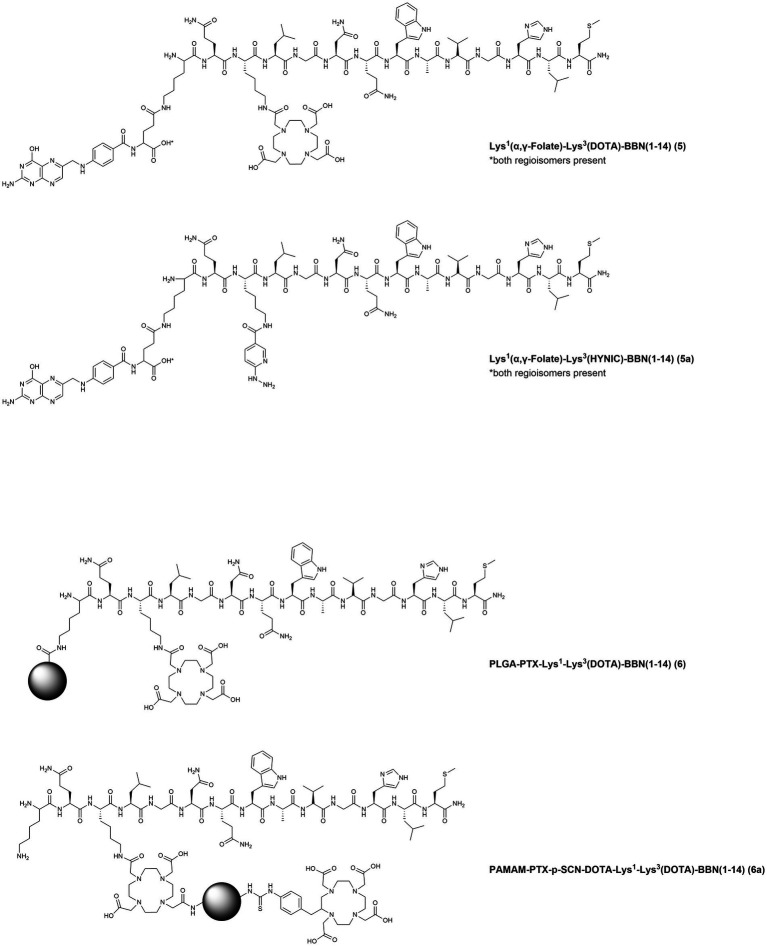
Chemical structures of the GRPR-targeting agonists 5–7a, evaluated in preclinical studies upon labeling with suitable radionuclides.

**Figure 6:**
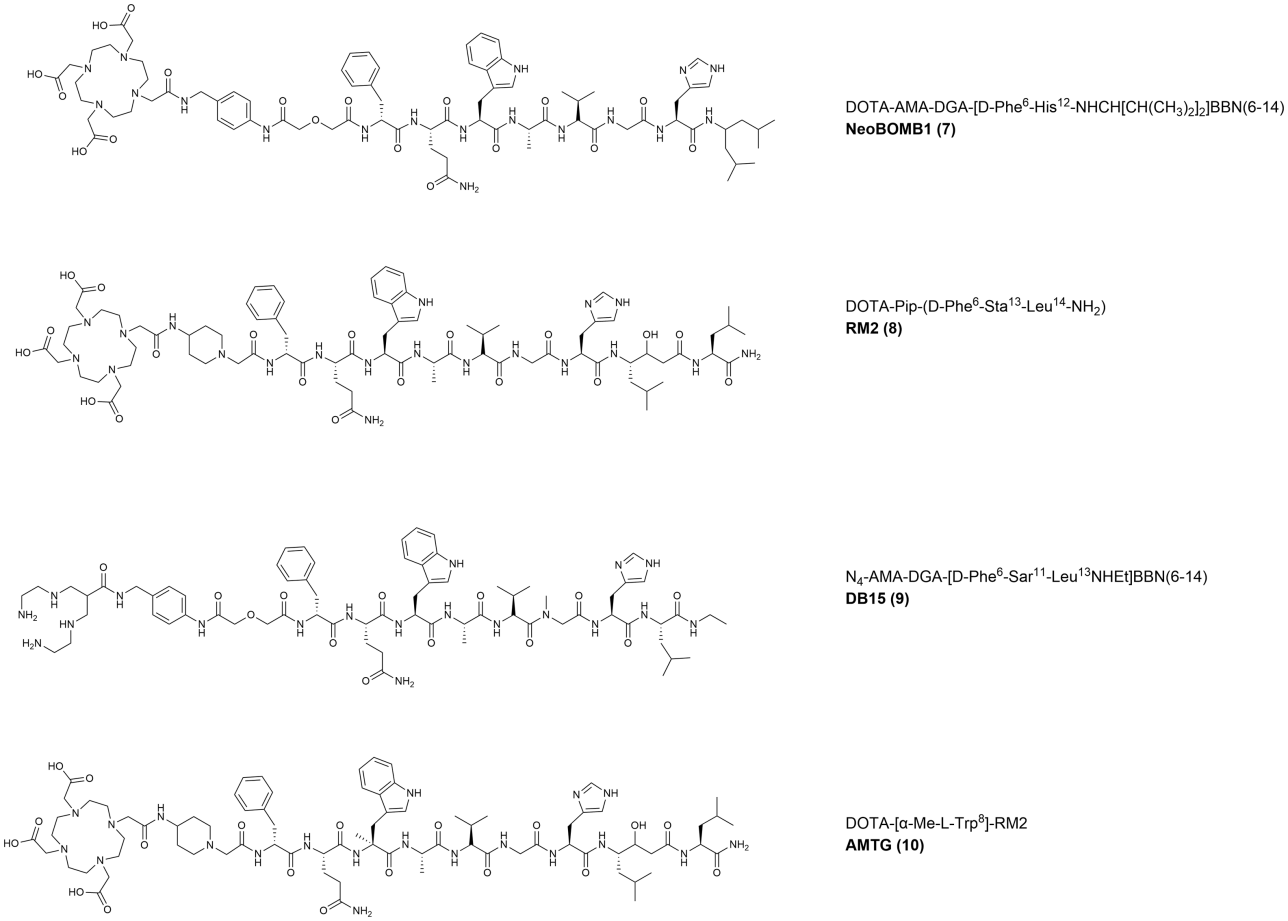
Chemical structures of the GRPR-targeting antagonists 8–11, evaluated in preclinical studies upon labeling with suitable radionuclides.

**Figure 7:**
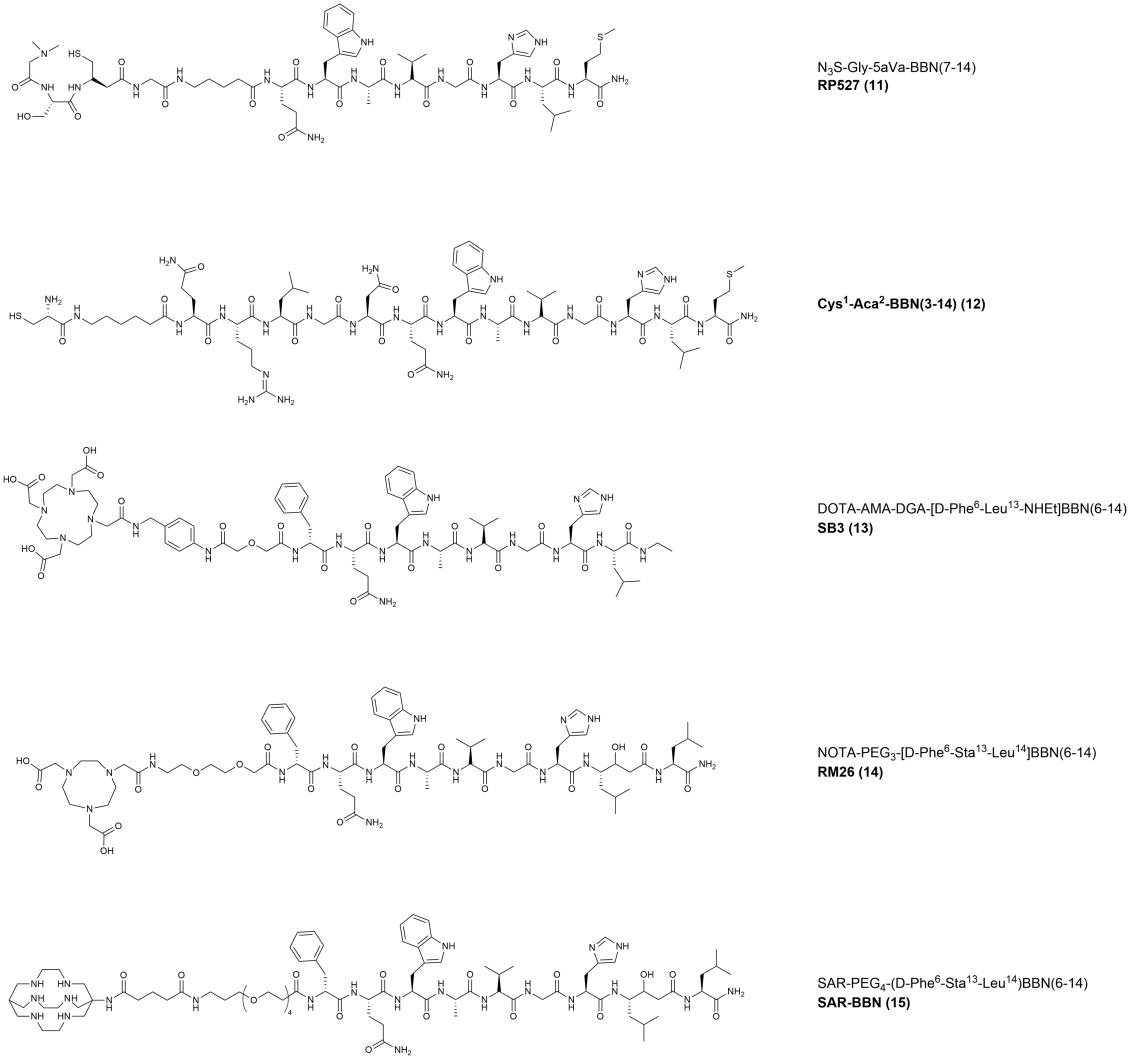
Chemical structures of the GRPR-targeting agonists 12–13 and antagonists 14–16, evaluated in clinical studies upon labeling with suitable radionuclides.

Several GRPR-targeting molecules, with either agonistic or antagonistic behavior, have been developed in the last decades by functionalization of their N-terminus with a variety of chelators and spacers and then radiolabeled with medically relevant radionuclides. Such modifications heavily influence the biological behavior of the final compounds and therefore deserve thorough preclinical and clinical investigations (76). In the next sections, we have gathered all GRPR radioligands that have been evaluated in BC preclinical models or in clinical settings. The chemical structures of the compounds and the corresponding numbers in the text are displayed in Figures 4–7 while the information about the compounds, relevant radionuclides used, intended use, phase of evaluation and references is resumed in Table 1.

**Table 1:** GRPR-radioligands evaluated in pre-clinical or clinical models of BC with the relevant radionuclide, modality and evaluation phase.

Compound	Behavior	Radionuclide	Modality	Evaluation phase	Ref.
1	Agonist	^64^Cu	PET	Pre-Clinical	(77)
2, 2a	Agonist	^64^Cu, ^68^Ga, ^18^F	PET	Pre-Clinical	(78)
3	Agonist	^64^Cu, ^68^Ga	PET	Pre-Clinical	(79)
4	Agonist	^111^In, ^68^Ga	PET	Pre-Clinical	(56, 66, 80)
5, 5a	Agonist	^177^Lu	SPECT	Pre-Clinical	(81)
6, 6a	Agonist	^177^Lu	Therapy, SPECT	Pre-Clinical	(82, 83)
7	Antagonist	^67^Ga, ^68^Ga, ^177^Lu	SPECT/PET, Therapy	Pre-Clinical, Clinical	(84–86)
8	Antagonist	^68^Ga	PET	Pre-Clinical, Clinical	(87–89)
9	Antagonist	^99m^Tc	SPECT	Pre-Clinical, Clinical	(90)
10	Antagonist	^177^Lu	Therapy, SPECT	Pre-Clinical	(91)
11	Agonist	^99m^Tc	SPECT	Clinical	(92, 93)
12	Agonist	^99m^Tc	SPECT	Clinical	(94)
13	Antagonist	^68^Ga	PET	Clinical	(95)
14	Antagonist	^68^Ga	PET	Clinical	(96)
15	Antagonist	^64^Cu	PET	Clinical	(97)

### 5.1. Preclinical studies with GRPR-targeting radiopeptides: agonists

In a study from 2007, the bombesin truncated octapeptide BBN(7–14) was conjugated to a DOTA chelator through linkers of different lengths (4, 5, 6, 8, and 12 carbons) and evaluated in a GRPR-expressing BC preclinical model. Upon radiolabeling with copper-64, the derivatives demonstrated promising *in vitro* and *in vivo* results. In particular, the compound DOTA-Aoc-BBN(7–14) (Figure 4, **1**), bearing the 8-carbons linker (Aoc: 8-Aminooctanoic Acid), showed an IC_50_ value in the low nanomolar range (6.7 ± 1.1 nM) and a quick internalization (1,419 ± 109 fmol/mg after 4 h) in the human BC cell line T47D, that expresses both GRPR and ER. *In vivo* studies, performed in female SCID mice bearing T47D xenografts, revealed a rapid blood clearance and high GRPR-mediated uptake in the pancreas and tumor, with the tumors being clearly visualized by microPET imaging studies (77).

In 2009, heterodimeric RGD-BBN peptidic analogues, containing an RGD (cyclo(Arg-Gly-Asp-D-Tyr-Lys) and a BBN(Aca-BBN(7–14), Aca: 6-Aminocaproic Acid) moieties were designed for the dual targeting of GRPR and integrin α_v_β_3_, a protein involved in cancer angiogenesis. The heterodimers were functionalized first with a p-SCN-Bn-NOTA chelator (2-S-(4-Isothiocyanatobenzyl)-1,4,7-triazacyclononane-1,4,7-triacetic acid) to afford NOTA-RGD-BBN(7–14) (Figure 4, **2**) for the radiolabeling with the positron emitting radionuclides copper-64 and gallium-68. Then, the heterodimer was also functionalized with a pegylated spacer (PEG_3_-RGD-BBN(7–14) (Figure 4, **2a**) and radiolabeled with fluorine-18 using the compound N-succinimidyl-4-^18^F-fluorobenzoate ([^18^F]-SFB) as the synthon. The radiotracers were evaluated for their ability to detect BC by microPET imaging using two orthotopic BC models, T47D (positive GRPR expression and low integrin α_v_β_3_ expression) and MDA-MB-435 (GRPR-negative expression and high integrin α_v_β_3_ expression). The different prosthetic labeling groups, as well as the different chelators and isotopes, resulted into different tumor targeting properties and *in vivo* pharmacokinetics. The radiofluorinated compound displayed the lowest tumor uptake but the highest contrast, due to the rapid washout of the tracer from blood and normal organs. On the other hand, the radiometalled compounds showed higher tumor accumulation and higher background uptake, with the ^64^Cu-radiolabeled compound having the highest retention in the liver and kidneys (78).

In 2012, a potent agonist with high GRPR affinity was developed by several modifications of the BBN amino acid sequence. This derivative, NOTA-PEG-[D-Tyr^6^-βAla^11^-Thi^13^-Nle^14^]BBN(6–14) (Figure 4, **3**), was radiolabeled with copper-64 and gallium-68 and preclinically evaluated in BC and PC models. The choice of the metal and the charge of the resulting metal complexes influences GRPR affinity, as demonstrated by the inhibitory constants (K_i_) values. The non-metallated peptide NOTA-PEG-BBN(6–14) and the corresponding Cu(II) and Ga(III) complexes had K_i_ values of 1.27 ± 0.95, 1.60 ± 0.59 and 4.87 ± 1.27 nM, respectively, as determined using the BC cell line T47D. Despite the different affinities, the two radiotracers exhibited similar cell uptake after incubation with T47D cells and with the human PC cell line PC3. Biodistribution studies in xenografts-bearing Balb/c nude mice confirmed a similar tumor uptake in the two cancer models and optimal *in vivo* stability, highlighting these molecules as promising candidates for GRPR PET-imaging of BC and PC (79).

In a study from 2015, the potent bombesin agonist AMBA [DOTA-Gly-4-aminobenzoyl-BBN(7–14), (Figure 4, **4**)], obtained by conjugation of the BBN(7–14) moiety to the universal chelator DOTA using a glycine 4-aminobenzoyl spacer, was radiolabeled with indium-111. The tracer was then used for autoradiography studies and to screen nine human BC cell lines *in vitro* (SUM44PE, MCF7, T47D, UACC812, BT474, CAMA-1, SUM52PE, HCC1806, Hs578t) with regard to their GRPR expression by cell uptake/internalization studies. Furthermore, 50 clinical specimens of BC, with known ER status, were also tested in the same experimental setup in order to identify those with the highest GRPR-specific uptake. Almost all (96%) human BC specimens were found to express GRPR, with the majority (56%) of the samples showing high GRPR-expression (above 75%, as scored visually by 3 independent observers). Six of the nine BC cell lines were also GRPR-positive, with the highest uptake values found in the cell lines T47D and MCF7 (approximately 10 and 4% in relation to the total added dose, respectively). Additional analysis by RT-qPCR indicated a good correlation between [^111^In]In-AMBA uptake and GRPR mRNA expression (56).

Further investigations on the therapeutic potential of [^177^Lu]Lu-AMBA were performed by evaluating its *in vitro* cytotoxic effect. The incubation of T47D cells with 50 MBq of the radiotracer for a period of 4 h resulted in a significant (80%) reduction of the cell viability. However, in a phase I escalation study in metastatic castration-resistant prostate cancer (mCRPC) patients, several side effects due to the administration of therapeutic doses of [^177^Lu]Lu-AMBA were being reported, including severe abdominal cramps and emesis caused by the high gastrointestinal uptake, as previously mentioned (66). Therefore, the following *in vivo* studies were performed using the GRPR antagonist [^111^In]In-JMV4168, in mice bearing T47D and MCF7 xenografts (57). In both tumor models visualization of the lesions was achieved by microSPECT/CT imaging, however, the T47D tumors exhibited higher uptake, in agreement with the previous *in vitro* findings.

Another study published in 2015 compared, in terms of *in vivo* performance, the PET tracers [^68^Ga]Ga-AMBA and 2-[^18^F]FDG in a GRPR-expressing preclinical BC model. In particular, imaging studies to evaluate the tumor response to hormone therapy were performed before and after tamoxifen treatment in xenograft-bearing mice implanted with the ER-positive human BC cell line ZR75-1. While 2-[^18^F]FDG uptake was low and the tumor hardly visible over the background, the administration of [^68^Ga]Ga-AMBA led to a clear delineation of the tumors prior to treatment with a significantly lower uptake observed after therapy, indicating that the tracer could be useful for monitoring tumor shrinking during therapy (80). Two possible explanations have been proposed for the reduced uptake of the tracer after tamoxifen treatment: (1). A reduced tumor metabolic activity due to the diminished stimulation of the ER. (2). A possible role of ER on the modulation of GRPR expression by cancer cells, as previously reported for PC (98).

In 2016, two studies from the same group reported the development of the heterobivalent compound Lys^1^(α,γ-Folate)-Lys^3^-DOTA-BBN(1–14) (Figure 5, **5**) for theranostic applications of BC expressing both FR and GRPR. Folate is essential for the fast metabolism of cancer cells because of its pivotal role in DNA synthesis and repair; therefore, the concomitant target of FR and GRPR is expected to improve both the BC cells recognition and the theranostic properties of the tracer. In the first study, the compound was radiolabeled with lutetium-177, through the insertion of a DOTA moiety at the Lys^3^ residue. *In vivo* studies were conducted in athymic mice bearing T47D xenografts, since this cell line has been previously reported also for its high FR expression (99). The therapeutic radiotracer showed high tumor uptake (5.71 ± 0.58% I.A./g at 4 h p.i.) in athymic mice bearing T47D tumors and the lesions were clearly visualized by micro-SPECT/CT imaging (81). In the second study, the Lys^3^ was used to insert a HYNIC (hydrazinonicotinamide) moiety facilitating the radiolabeling with technetium-99 m and resulting in the compound Lys^1^(α,γ-Folate)-Lys^3^-HYNIC-BBN(1–14) (Figure 5, **5a**). *In vitro* and *in vivo* studies showed specific uptake in T47D cells (38.27 ± 0.91% of total activity) and xenografts (5.43 ± 0.93% I.A./g at 4 h p.i.). Moreover, when compared to the respective monomers [^99m^Tc]Tc-Folate and [^99m^Tc]Tc-Bombesin, improved imaging properties were demonstrated (standardized uptake values in the tumors of 1.38 ± 0.33, 0.53 ± 0.11, and 0.86 ± 0.17 for the heterobivalent compound, the [^99m^Tc]Tc-Folate and the [^99m^Tc]Tc-Bombesin, respectively) (100).

In 2019, poly(D,L-Lactide-co-Glycolide) acid (PLGA) nanoparticles were functionalized with the chemotherapic drug paclitaxel (PTX) and the compound Lys^1^-Lys^3^-DOTA-BBN(1–14), to obtain the targeted controlled-release nanodrug PLGA-PTX-Lys^1^-Lys^3^(DOTA)-BBN(1–14) (Figure 5, **6**) for concomitant radiotherapy and chemotherapy of BC. After radiolabeling with lutetium-177, *in vitro* studies demonstrated specific uptake and high synergic cytotoxic effect in the human TNBC cell line MDA-MB-231. Moreover, microSPECT/CT imaging studies allowed not only the visualization of subcutaneous MDA-MB-231 tumors but also of pulmonary MDA-MB-231 micrometastases (82). The same group, shortly after, also reported on the development of a different nanosystem based on the polyamidoamine (PAMAM) dendrimer, functionalized with PTX and Lys^1^-Lys^3^-DOTA-BBN(1–14), PAMAM-PTX-p-SCN-DOTA-Lys^1^-Lys^3^-DOTA-BBN(1–14) (Figure 5, **6a**). *In vitro* and *in vivo* cellular studies were performed upon ^177^Lu-radiolabeling using the T47D BC cell line and demonstrated a selective and synergic radio-chemotherapeutic effect and excellent tumor visualization by microSPECT/CT imaging (83).

### 5.2. Preclinical studies with GRPR-targeting radiopeptides: antagonists

In 2017, the GRPR-antagonist NeoBOMB1 (DOTA-AMA-DGA-[D-Phe^6^-His^12^-NHCH[CH(CH_3_)_2_]_2_]-BBN(6–14) (Figure 6, **7**)), that had already shown promising preclinical and clinical results as diagnostic and therapeutic probe for PC, was also evaluated in a BC preclinical model (84). The BBN(6–14) motif was modified to display an antagonist behavior by truncation of the C-terminal Met and ethylamidation of the Leu^13^, while the Asn^6^ residue was substituted with a D-Phe to increase the potency. The BBN antagonist was then functionalized, via a p-aminomethylaniline-diglycolic acid spacer (AMA-DGA), with the DOTA chelator to generate the compound NeoBOMB1. The tracer, upon radiolabeling with gallium-67, showed high GRPR affinity (IC_50_ of 2.2 ± 0.2 nM), high uptake in T47D cells (58% after 2 h incubation), as well as high and specific tumor and pancreas uptake in T47D xenografts (8.67 ± 2.88 and 36.86 ± 3.58% IA/g, respectively at 4 h p.i.) (85).

**Figure 8:**
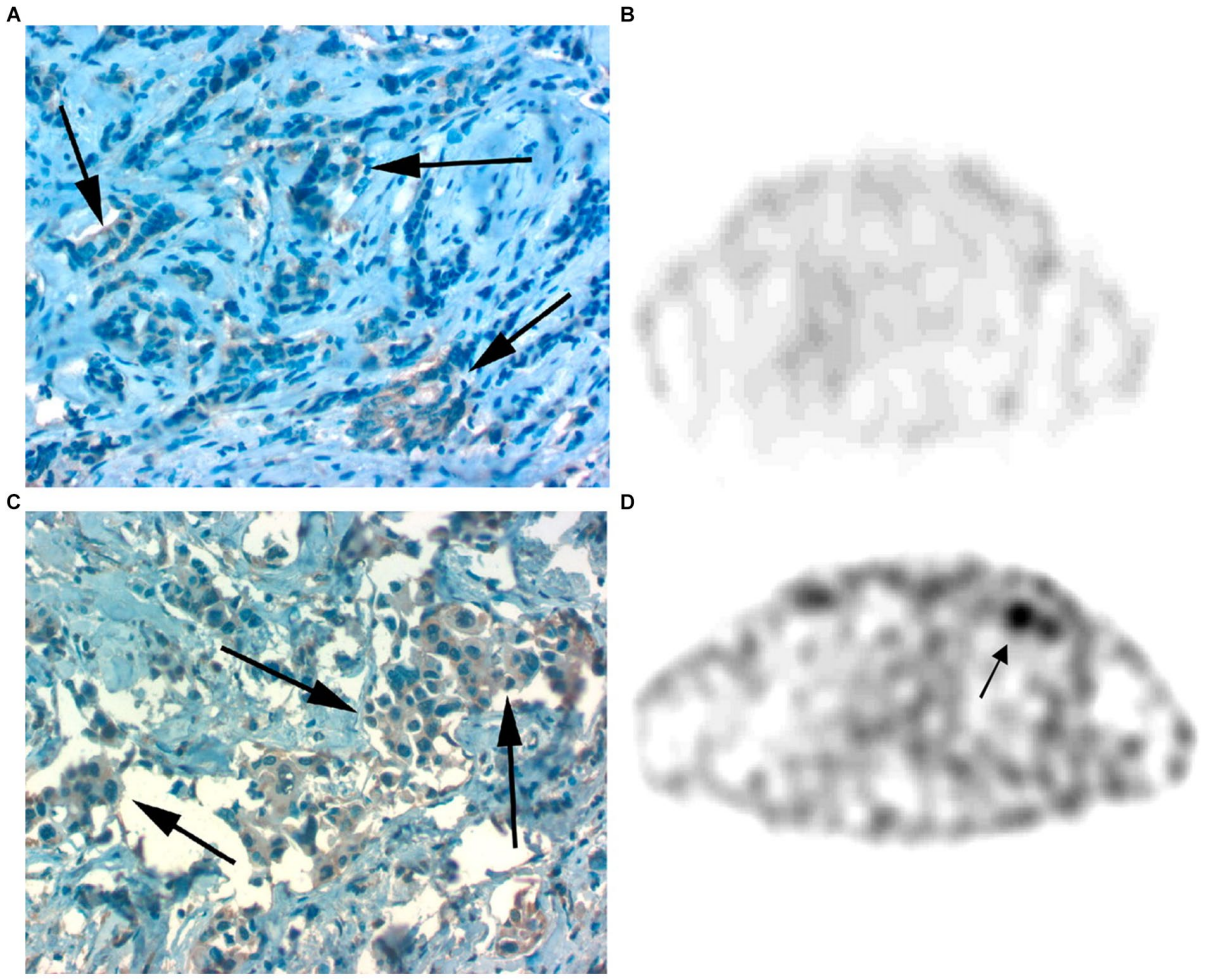
**(A,B)** Faint GRPR staining of BC cells as detected by IHC **(A)** and lack of [^99m^Tc]Tc-RP527 uptake in SPECT scan from the same patient **(B)**. **(C,D)** Pronounced GRPR staining of BC cells as detected by IHC **(C)** and high uptake of [^99m^Tc]Tc-RP527 in SPECT scan from the same patient **(D)**. Reprinted from Gastrin-Releasing Peptide Receptor Imaging in Human Breast Carcinoma Versus Immunohistochemistry by Van de Wiele et al. (93), © SNMMI.

In 2019, the statine-based potent antagonist RM2 (DOTA-Pip-[D-Phe^6^-Sta^13^-Leu^14^]BBN(6–14) (Figure 6, **8**)), bearing a piperidine spacer and the chelator DOTA, was radiolabeled with gallium-68 and its efficiency to positively stain 14 primary BC samples from patients that did not receive any neoadjuvant treatment was evaluated (10 primary tumors and 4 metastatic lymph nodes) (87). When compared to 2-[^18^F]FDG, the uptake of [^68^Ga]Ga-RM2 quantified by tissue micro-imaging was significantly higher (45.31 ± 13.23 vs. 16.51 ± 28.45% binding) and displayed a complementary pattern in ER-positive tumor samples. These results suggest that GRPR targeting might be a valid alternative to 2-[^18^F]FDG for the imaging of ER-positive tumors with potential also for targeted radionuclide therapy in patients with progressive metastatic disease following conventional treatments (88).

**Figure 9:**
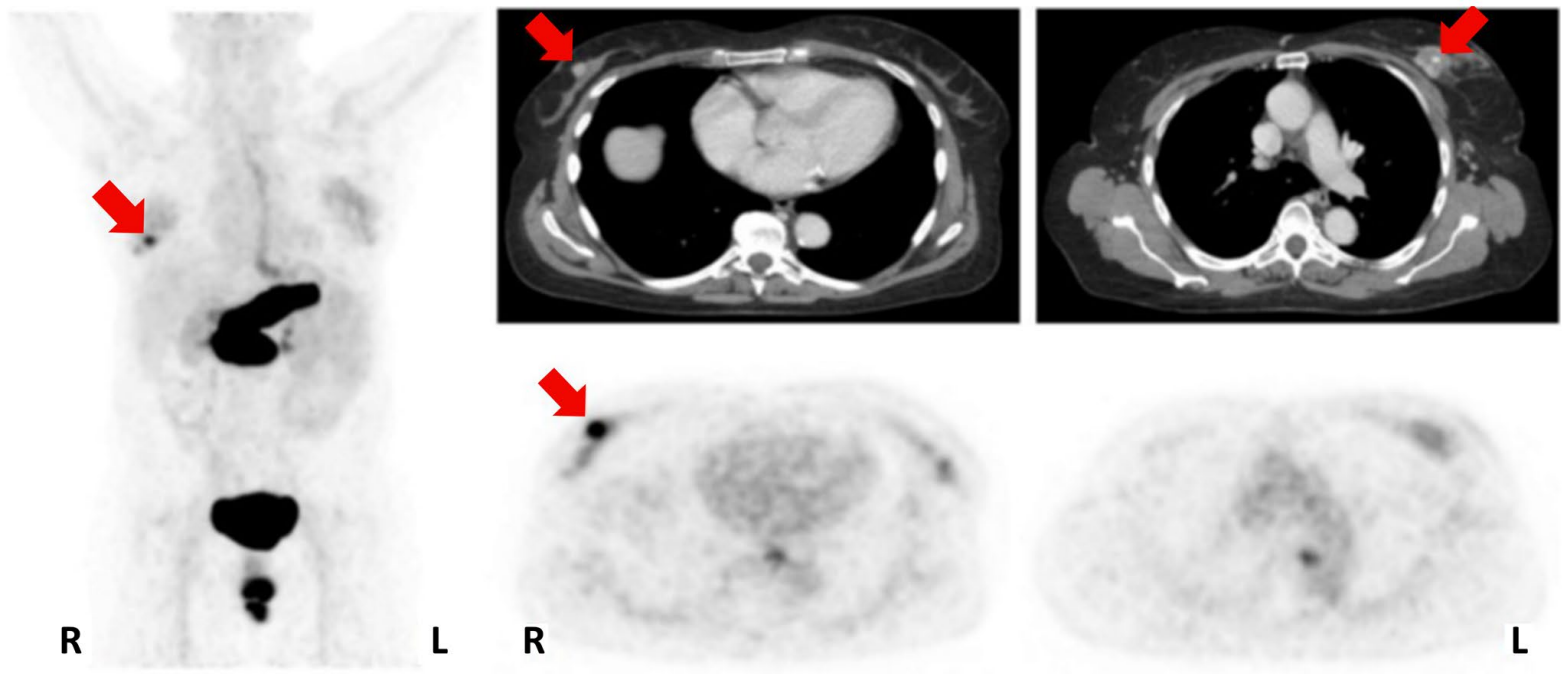
Patient with an ER/PR-positive tumor in the right breast (PET-positive; SUVmax 8.32) and an ER/PR-negative tumor on the left side (PET-negative; SUVmax 2.68). MIP left; CT upper row; [^68^Ga]Ga-RM2-PET lower row; primary tumors indicated by red arrows. Reprinted from Gastrin-releasing Peptide Receptor Imaging in Breast Cancer Using the Receptor Antagonist 68Ga-RM2 and PET by Stoykow et al. (89), under Creative Commons Attribution (CC BY-NC) license.

In 2021, the novel tracer [^99m^Tc]Tc-DB15 (N_4_-AMA-DGA-[D-Phe^6^-Sar^11^-Leu^13^-NHEt]BBN(6–14) (Figure 6, **9**)), based on the des-Met antagonists series, was developed. The peptide was further modified by substituting the Gly^11^ with a sarcosine residue (N-methylglycine, Sar) to increase its metabolic stability and then functionalized, through a AMA-DGA linker, with an acyclic tetraamine chelator (N_4_: 6-carboxy-1,4,8,11-tetraazaundecane) for the radiolabeling of the SPECT emitter technetium-99 m. The tracer was evaluated in two GRPR-expressing preclinical models of BC and PC and displayed high GRPR affinity (IC_50_ = 0.37 ± 0.03 nM) and high specific uptake (24.2 ± 0.7% after 30 min incubation at 37°C) in the T47D cell line. In T47D xenografts, the radiotracer showed high metabolic *in vivo* stability and prolonged GRPR-specific uptake (14.01 ± 2.87% IA/g at 1 h p.i. and 7.55 ± 1.81% IA/g at 24 h p.i. in the tumor and 130% IA/g at 1 h p.i. and approx. 2% IA/g at 24 h p.i. in the pancreas) (90).

**Figure 10:**
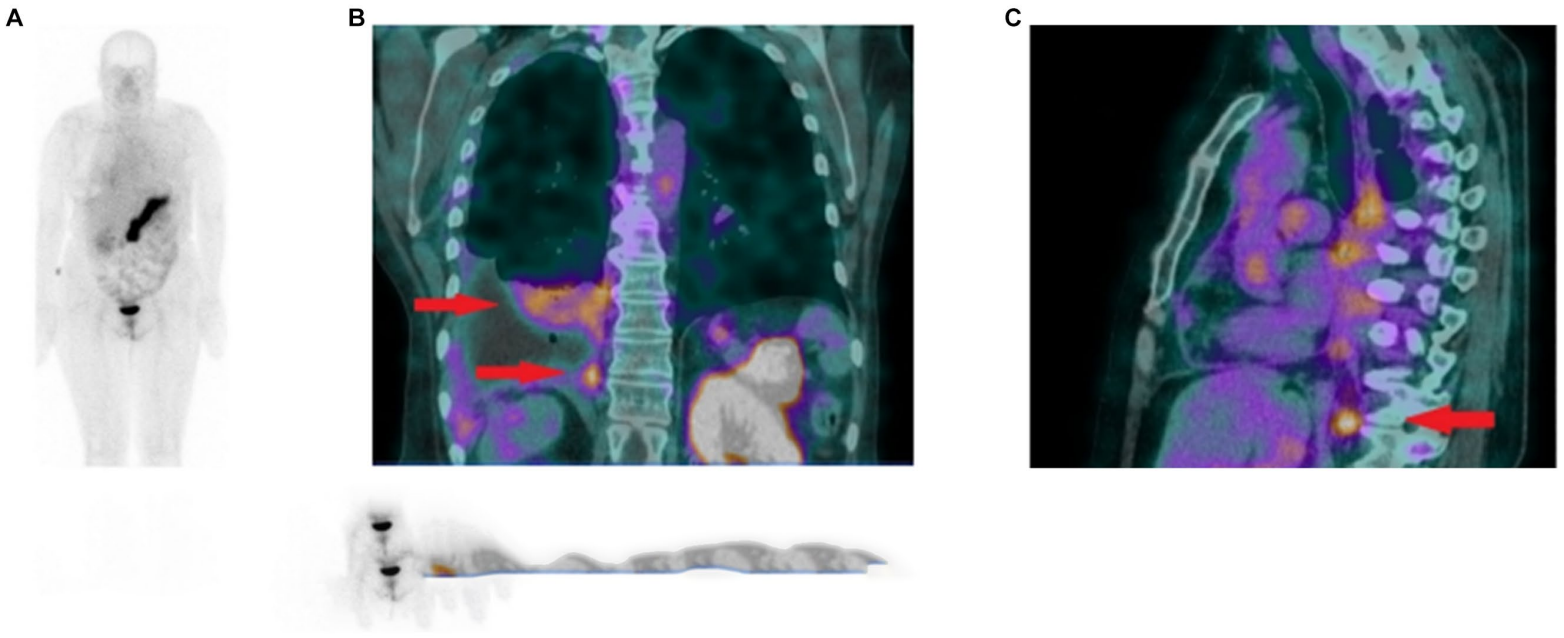
**(A)** Whole body scan obtained 3 h after injection of [^99m^Tc]Tc-DB15 in the anterior projection shows physiological accumulation in the pancreas and increased uptake in the right pleura. **(B)** SPECT/CT coronal image of the chest presenting increased tracer uptake in the metastatic infiltrations in the pleura and lung parenchyma (red arrows). **(C)** SPECT/CT sagittal image of the chest depicting increased radioactivity accumulation in an enlarged (metastatic) phrenic lymph node (red arrow). Adapted from [99mTc]Tc-DB15 in GRPR-Targeted Tumor Imaging with SPECT: From Preclinical Evaluation to the First Clinical Outcomes by Nock et al. (90), under Creative Commons Attribution (CC BY) license.

The GRPR-antagonists RM2 and NeoBOMB1 are characterized by a short *in vivo* half-life, due to the presence of cleavage sites that are susceptible to the action of the neutral endopeptidases. Therefore, to obtain derivatives with enhanced *in vivo* stability, a novel GRPR antagonist was developed in 2022 by substituting the Trp^8^ of RM2 with a α-methyl-L-tryptophan residue (α-Me-L-Trp) (91, 101). The peptide was then conjugated, via a piperidine spacer, to the DOTA/DOTAGA chelators resulting in the novel derivatives AMTG/AMTG2 respectively, which were used for the generation of the relevant therapeutic radiotracers after their radiolabeling with lutetium-177. The tracer [^177^Lu]Lu-AMTG (Figure 6, **10**) showed promising preclinical results, comparable with [^177^Lu]Lu-RM2 in terms of GRPR affinity, internalization rate and lipophilicity. A considerably higher *in vitro* and *in vivo* stability was observed, together with a 35% higher tumor retention and slower clearance in PC3 xenografts. The IC_50_ values of ^nat^Lu-AMTG in PC3 and T47D cells (3.0 ± 0.1 and 1.0 ± 0.1 nM, respectively) indicate high GRPR affinity that warrant additional studies in BC preclinical *in vivo* models.

### 5.3. Clinical studies with GRPR-targeting Radiopeptides: Agonists

**Figure 11:**
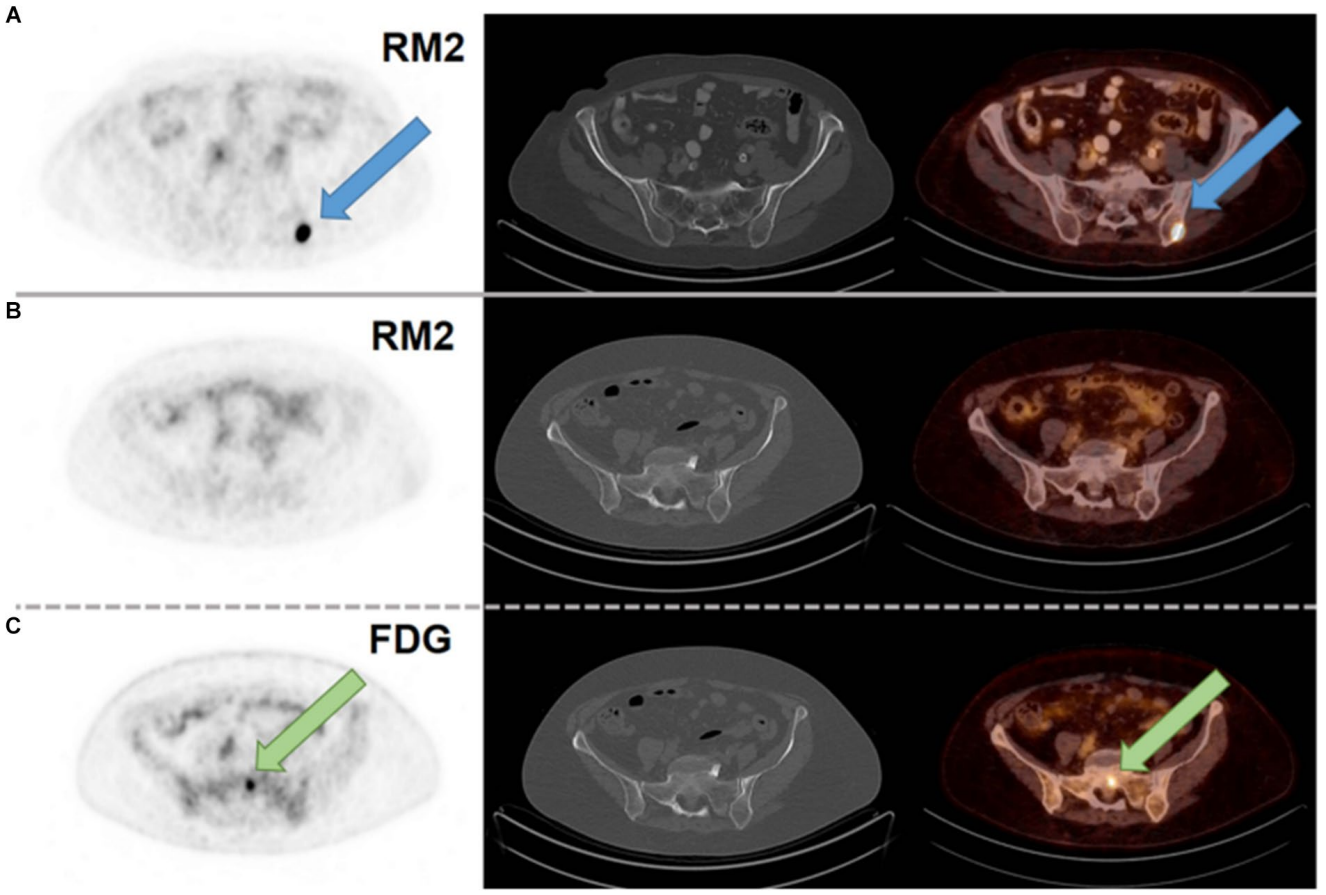
**(A)** [^68^Ga]Ga-RM2 PET/CT of a patient with a metastasis in the left iliac bone with intense RM2 binding and not seen on CT (blue arrow; SUVmax 32.1). **(B)** [^68^Ga]Ga-RM2 PET/CT of a patient with a bone metastasis in the sacrum without RM2 binding and not seen on CT (SUVmax 1.5; second row). **(C)** [^18^F]FDG PET/CT of the same patient with intense hypermetabolism in the sacrum (green arrow; SUVmax 5.8; third row). Axial slices of PET scans (first column), CT scans (second column) and fusion images (third column). Reprinted from Gastrin-Releasing Peptide Receptor Antagonist [68Ga]RM2 PET/CT for Staging of Pre-Treated, Metastasized Breast Cancer by Michalski et al. (102), under Creative Commons Attribution (CC BY) license.

In 2001, the radiotracer [^99m^Tc]Tc-RP527 (Figure 7, **11**) containing a tripeptide composed by a dimethylated glycine, a L-serine and an acetamidomethyl L-cysteine (dmGly-L-Ser-Acm-L-Cys), and the N_3_S chelator for the radiolabeling with technetium-99 m, was developed. By losing the acetamidomethyl protecting group, the chelator forms a stable complex with [^99m^Tc]Tc(V)O and is linked, through a Gly-5-Aminopentanoic acid (gly5aVa) spacer, to the N-terminus of BBN(7–14). [^99m^Tc]Tc-RP527 was tested in patients with BC and showed specific tumor uptake and good imaging characteristics in four out of six patients screened (92).

In 2008, the same radiotracer was used for scintigraphy in 9 patients with clinical diagnosis of BC and 5 patients with tamoxifen-resistant bone-metastasized BC. The results from the scans were compared with routine staging examinations, routine histology and IHC analysis, as shown in Figure 8. The primary tumors were all GRPR positive and [^99m^Tc]Tc-RP527 uptake was evident in 8 out of 9 patients. The involved lymph nodes were also clearly visualized in the patients with positive uptake, while in 1 of the patient with osseous metastasis only half of the lesions were visualized. In the patients with tamoxifen-resistant osseous metastasis, no uptake of the tracer was observed (93).

**Figure 12:**
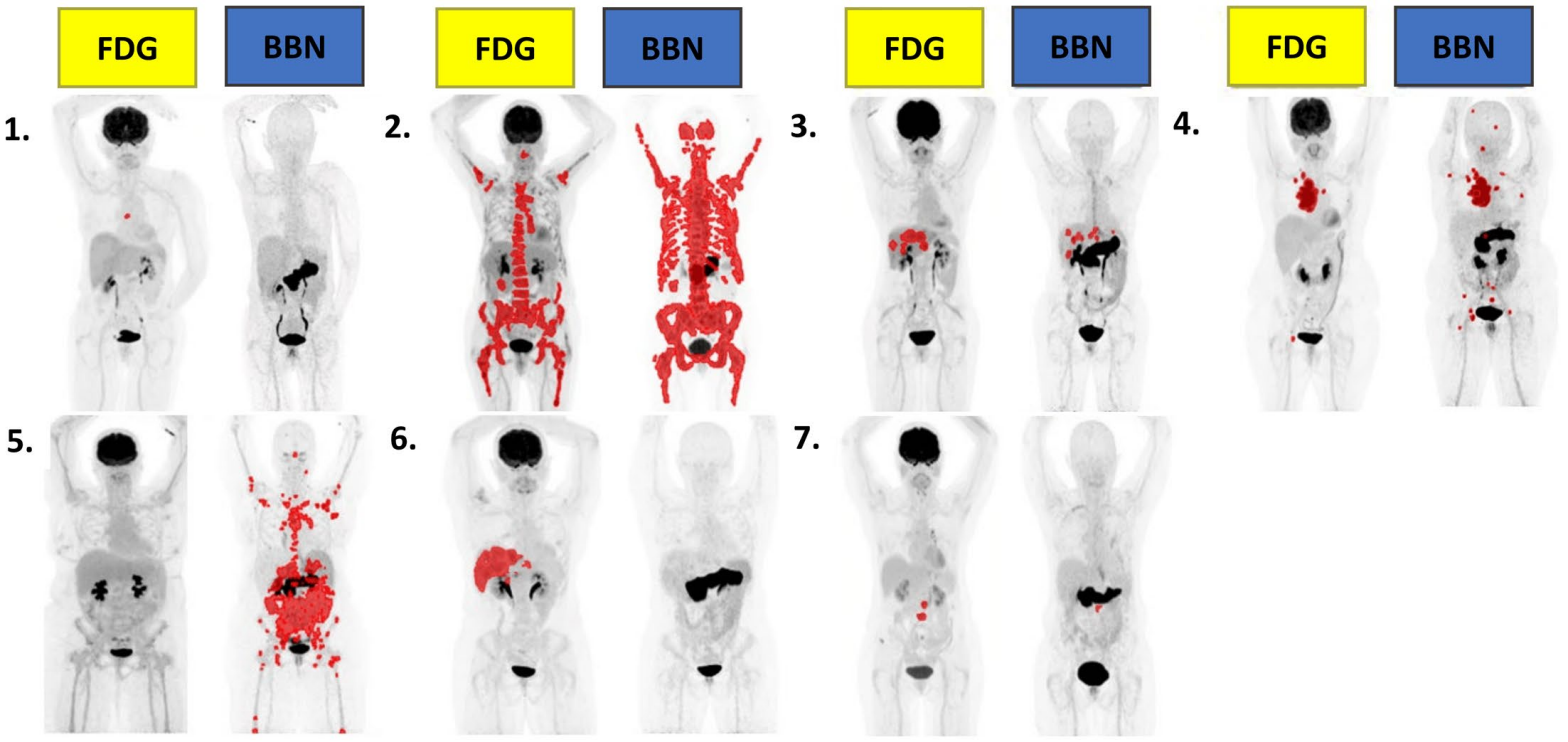
Representative MIPs of total tumor volume quantitation from all 7 patients using MIM software using a threshold of SUVmax>3. BBN quantitation performed on 1h post-injection acquisition. Adapted from 64Cu-SAR-Bombesin PET-CT Imaging in the Staging of Estrogen/Progesterone Receptor Positive, HER2 Negative Metastatic Breast Cancer Patients: Safety Dosimetry and Feasibility In A Phase I Trial by Wong et al. (97), under Creative Commons Attribution (CC BY) license.

In 2002, in a pilot study performed in 3 patients with primary BC, the non-specific tracer [^99m^Tc]Tc-Sestamibi was compared with [^99m^Tc]Tc-BBN (Figure 7, **12**). The latter probe was obtained by modification of the N-terminus of BBN, by removal of the Glu residue and insertion of a cysteine and of a 6-Aminohexanoic acid (Aca) spacer for labeling with technetium-99 m. The GRPR-specific tracer showed higher contrast and IHC studies confirmed that [^99m^Tc]Tc-BBN was taken up selectively by metastatic cancer cells with no uptake in the lymph vessels, lymphocytes and inflammatory cells. The only lymph node present in the study appeared smaller and less active when imaged with [^99m^Tc]Tc-BBN, indicating that non-specific uptake mechanisms might influence the images obtained with [^99m^Tc]Tc-Sestamibi (94).

In 2007, the tracer [^68^Ga]Ga-AMBA (Figure 4, **4**) was assessed for the ability to image GRPR in 10 patients, including 2 BC patients. After administration of the tracer (25–50 μg peptide/dose) several pathological lesions including lymph nodes, liver and bone metastasis were visualized and [^68^Ga]Ga-AMBA proved to be a valuable tool to assess GRPR tumor expression status. The administration was tolerated with minor adverse effects and the tracer had fast renal clearance and high uptake mainly in pancreas, intestine and esophago-gastric junction. Nonetheless, shortly after, a phase I escalation study using [^177^Lu]Lu-AMBA in mCRPC patients was ended prematurely because of the severe side effects (66).

### 5.4. Clinical studies with GRPR-targeting radiopeptides: antagonists

Meanwhile, studies conducted with radiolabeled somatostatin antagonists had already demonstrated the superior *in vivo* tumor targeting properties and better tolerability of radiolabeled antagonists over agonists. Therefore, this paradigm shift toward the use of antagonists was quickly extended also to radioligands directed at other peptide receptors, including GRPR.

In 2015, the radioantagonist [^68^Ga]Ga-SB3 (DOTA-AMA-DGA-[D-Phe^6^-Leu^13^-NHEt]BBN(6–14) (Figure 7, **13**)) was evaluated in a preclinical PC model. The tracer displayed good *in vivo* stability and high, specific and prolonged retention in PC3 xenografts. The novel tracer was then administered into 17 patients with advanced PC or BC. Despite the lack of evaluation in a BC preclinical model, [^68^Ga]Ga-SB3 elicited no adverse effects and allowed clear visualization of cancer lesions in 4 of the 8 BC patients involved (95).

In 2016, [^68^Ga]Ga-RM2 (Figure 6, **8**) was used for pre-treatment staging by PET/CT imaging of patients with primary BC, as shown in Figure 9. The study revealed a low to moderate uptake of the tracer in normal breast tissue, while the tumor uptake correlated well with ER/PR expression, HER2 status and MIB-1 proliferation index. A strongly increased uptake of the tracer was observed in 13 of the 18 tumors analyzed and all the PET-positive tumors stained positively also for ER and PR. Importantly, high [^68^Ga]Ga-RM2 uptake was detected also in lymph nodes and bone metastasis, confirming that ER expression is a good predictor for GRPR expression quantification by [^68^Ga]Ga-RM2 PET (89).

In 2018, a prospective pilot study investigated the value of [^68^Ga]Ga-NOTA-RM26 (Figure 7, **14**), a GRPR antagonist bearing the same statine-based amino acid sequence as RM2, the chelator NOTA and a short pegylated linker (PEG_3_). In 35 women with suspect of BC, [^68^Ga]Ga-NOTA-RM26 demonstrated significantly enhanced uptake in ER-positive BC. Furthermore, the study also revealed that tracer uptake on normal breast tissues was correlated to the menstrual status of the patients, with higher values during the secretory phase (96).

In 2021, the GRPR-targeting radioantagonist [^99m^Tc]Tc-DB15 (Figure 6, **9**) showed promising results in a pilot translational study in two advanced BC patients. The tracer allowed the visualization, by SPECT/CT imaging, of disseminated bone metastasis, soft tissues metastasis and lymph nodes that warrant further investigations, as shown in Figure 10 (90).

Another study reported in 2021, describes the use of [^68^Ga]Ga-RM2 (Figure 6, **8**) for PET/CT tumor visualization in patients with pre-treated, ER-positive BC and suspected metastases. The pilot study included 8 patients with initial ER-positive and pre-treated BC. Seven of the 8 patients were still in treatment with endocrine therapy. In 6 patients, a strong tracer uptake was observed in all metastatic lesions while no uptake was observed in the other two patients, as shown in Figure 11. These results suggest that [^68^Ga]Ga-RM2 imaging could support treatment decision in the majority of patients with advanced disease stage of pre-treated ER-positive BC (102).

## 6. BC clinical trials with GRPR-radioligands

In 2019, the Phase II clinical trial NeoFIND (NCT03724253) evaluated the tracer [^68^Ga]Ga-NeoBOMB1 (Figure 6, **7**) in 19 patients with advanced GRPR-expressing malignancies, including BC. The study confirmed the safety profile of [^68^Ga]Ga-NeoBOMB1 and further provided whole-body dosimetry data, in the view of a possible therapeutic translation using [^177^Lu]Lu-NeoBOMB1. [^68^Ga]Ga-NeoBOMB1 uptake led to delineation of at least one primary or metastatic lesion in 17/19 patients and, in 9 patients, at least half of the primary or metastatic lesions that were detected with conventional imaging (e.g., MRI, 2-[^18^F]FDG PET) were also positive after the administration of [^68^Ga]Ga-NeoBOMB1. Two BC patients in the dosimetry subgroup had effective whole-body doses of 0.0203 and 0.0151 mSv/MBq, in agreement with previous dosimetry data obtained by the administration of the same tracer to patients with gastrointestinal stromal tumors (MITIGATE, EudraCT 2016–002053-38). [^68^Ga]Ga-NeoBOMB1 was well tolerated, with no related adverse event and detected different tumor types, although tumor-specific uptake was variable. Despite these encouraging results, the study was prematurely ended after enrolling 19 subjects due to claimed difficulties in enrolling patients for a diagnostic study (103).

Nonetheless, a Phase I/II clinical trial to evaluate the safety, tolerability, pharmacokinetics as well as the distribution, radiation dosimetry and anti-tumor activity of [^177^Lu]Lu-NeoBOMB1 in patients with positive [^68^Ga]Ga-NeoBOMB1 uptake is currently on-going and is expected to provide also additional data on the PET tracer (NeoRay, NCT03872778) (86).

A Phase I/II study from 2022 assessed the safety and potential of [^64^Cu]Cu-Sarcophagine-Bombesin ([^64^Cu]Cu-SAR-BBN) (Figure 7, **15**) PET/CT in re-staging metastatic BC with positive ER/PR expression and negative HER2 expression. The tracer is based on a sarcophagine derived chelator (MeCOSar: 5-(8-methyl-3,6,10,13,16,19-hexaaza-bicyclo[6.6.6]icosan-1-ylamino)-5-oxopentanoic acid) conjugated through a pegylated spacer (PEG_4_) to the potent statine-based GRPR antagonist [D-Phe^6^-Sta^13^-Leu^14^]BBN(6–14) (104). In the 7 patients enrolled, 6 with recurrent metastatic disease and 1 with de-novo metastatic BC, no adverse events were reported. GRPR status was assessed by IHC in available biopsies and staging with conventional imaging ([^18^F]FDG, bone scan and diagnostic CT) was carried out within 3 weeks before administration of the tracer. Six out of 7 patients were [^18^F]FDG positive while 5 were [^64^Cu]Cu-SAR-BBN positive; in the 4 patients positive with both tracers, higher uptake and higher avidity were observed with [^64^Cu]Cu-SAR-BBN, as shown in Figure 12. Dosimetry calculations estimated a whole-body effective dose of 1.9 mSv for 200 MBq injection of the tracer (97).

At the end of 2022, a new Phase I open-label has started to determine the safety and effectiveness of [^212^Pb]Pb-DOTAM-GRPR1 in subjects with various GRPR-expressing tumors, including BC patients (NCT05283330) (105). The tracer is based on the structure of a GRPR antagonist (no further information is given), linked to the chelator DOTAM (2-[4,7,10-tris(2-amino-2-oxoethyl)-1,4,7,10-tetrazacyclododec-1-yl]acetamide) for radiolabeling with the therapeutic alpha-emitter nuclide lead-212. Once the recommended Multiple Ascending Dose (MAD) dose will be determined, the subjects will be treated with up to 4 cycles of [^212^Pb]Pb-DOTAM-GRPR1, administered every 8 weeks.

## 7. Discussion

Radiopharmaceuticals are experiencing a new golden era with a substantial growth of their commercial market and the recent FDA approval of the therapeutic drugs Lutathera® ([^177^Lu]Lu-DOTATATE) and Pluvicto® ([^177^Lu]Lu-PSMA-617) for the treatment of neuroendocrine tumors and PC, respectively (106, 107). Their commercial success is due not only to advancements on the scientific and technological side but also to the consistent investments of several pharmaceutical companies in the field (108).

Beside the somatostatin receptor and the PSMA, GRPR is a highly promising target for the development of novel theranostic compounds, due to the high-density expression in several human cancers and the relatively low physiological expression in healthy tissues. In the last decades, several molecules derived from the endogenous ligand GRP have been developed to image and treat GRPR-expressing malignancies. However, the use of GRPR agonists have raised drug safety concerns that finally hampered their translation to clinical practice, despite some encouraging initial results obtained both in the preclinical and clinical settings (76). GRPR antagonists, in contrast, appear to combine optimal tumor targeting properties with improved pharmacokinetics and a better safety profile, which prompted the development of several molecules with translational potential.

The massive GRPR overexpression in neoplastic prostate tissues and the contemporary lack of GRPR in normal prostate tissues has led in the last years to the development of several GRPR-based radiotracers for imaging and therapy of PC (109, 110). Nonetheless, a considerable body of evidence also demonstrates that GRPR is a promising and relevant target also for BC, in particular for the luminal subtypes with ER-positive expression. In fact, a high density of GRPR has been found in the 83% of ER-positive and 12% of ER-negative BC luminal tumors (59). In these BC subtypes, GRPR expression is particularly encouraging, since it has been found not only in primary tumors but also in lymph nodes and distant metastasis (52, 58).

The significant correlation between GRPR and ER expression has been demonstrated in previous studies conducted in both BC and PC, suggesting a potential important role of ER in mediating GRPR expression and contributing to cancer development, nevertheless this still requires further investigations (54, 56, 98). However, even after anti-hormonal therapy, which can lead to a state of androgen/estrogen independence and loss of ER expression, imaging of GRPR with the tracer [^68^Ga]Ga-SB3 led to approximately 50% of positive scans in patients that already underwent previous therapies (95).

Ubiquitous GRPR expression has been observed also in normal breast tissues, where the function of GRP in breast physiology is still unclear. Furthermore, GRPR expression is apparently also correlated with the menstrual status and an enhanced uptake of the GRPR-targeting tracer [^68^Ga]Ga-NOTA-RM26 has been observed during the secretory phase (55, 96). Still, a high percentage of neoplastic breast tissues expresses GRPR in higher density and many GRPR antagonists developed in the last years have shown promising preclinical data that have encouraged their early clinical translation, in particular NeoBOMB1, RM2, DB15 and SAR-BBN. Such GRPR-targeting molecules, upon radiolabeling with clinically relevant nuclides, might have interesting applications in clinical practice, especially for a personalized staging strategy upon confirmation of ER-positivity in the biopsy or to monitor ER status over time, with important therapeutic and prognostic implications also for endocrine treatment or targeted radionuclide therapy (89).

The compounds share a similar peptide sequence and were obtained by introduction of the Sta^13^-Leu^14^ dipeptide at the terminal position of Bombesin (RM2 and SAR-BBN) or by removal of the Met^14^ and ethylamidation of the Leu^13^ residue (NeoBOMB1 and DB15). Several improvements are still possible toward the development of analogues with enhanced metabolic stability, higher affinity or by the use of new and more exotic radionuclides as far as they become accessible. In addition, at the preclinical stage, only a small part of the GRPR-targeting molecules developed so far has been evaluated in BC models. In the past few years, three-dimensional cell models from primary cell lines (single cells or co-cultures) and from patient-derived samples have gained popularity because of their closer representation of the *in vivo* tissues and pathology (89, 111). Despite the inherent limitations due to the complex nature of the tumor microenvironment, these models might provide a more accurate screening of radiopharmaceuticals at least in their initial *in vitro* evaluation and especially toward high-throughput screening and automation (112). The ER-positive BC cell line T47D was extensively used to establish *in vitro* and *in vivo* preclinical models, however, some differences in the *in vivo* methodology were observed. Only a minor number of the studies reported herein (3 out of 9) did not require estrogen supplementation for the xenografts to grow after cell implantation (81, 83, 100). In 3 of the remaining studies, 60-days slow release estrogen pellets were placed subcutaneously (77–79) while, in the other 3 studies, β-estradiol was added directly to the water supply at a concentration of 4 mg/L (56, 85, 90). Hormone supplementation, through 60-days slow release estrogen pellets was also used in the *in vivo* model using the human ER-positive BC cell lines ZR75-1 and MCF7 (56, 80). On the other hand, when using the human TNBC cell line MDA-MB-231 no hormone supplementation was used (82).

Upon radiolabeling with appropriate radionuclides, such as gallium-68, technetium-99 m, and copper-64, GRPR antagonists have proven their usefulness as PET/SPECT/CT tracers in several preclinical and clinical studies and have also demonstrated the advantages of using a targeted imaging approach over unspecific tracers, such as 2-[^18^F]FDG, for BC diagnosis, staging and re-staging (88). The availability of PET tracers to monitor the expression of crucial receptors such as ER, PR or HER2 played an important role to achieve accurate and non-invasive patient selection and stratification as well as improved therapeutic follow-ups (113). Nonetheless, the theranostic opportunity offered by radiopharmaceuticals is key to novel therapy options for BC patients, and PRRT based on GRPR-targeting radiopeptides is pivotal in this regard. In fact, since luminal tumors account for approximately 80% of all BC and a significant percentage is resistant or acquire resistance to hormone therapy, many patients are likely to benefit from the development of GRPR-radiotheranostics (51).

Moving forward, alpha-targeted therapy also offers good prospects because of the high linear energy transfer that makes them appealing for PRRT applications. Also, it is worth to mention that many alpha-emitters suitable for medical applications have a short half-life which is well matched with the short biological half-life and blood clearance of GRPR-targeting peptides. Nonetheless, careful dosimetry studies are needed to determine the maximum tolerated dose, taking into account their specific uptake in GRPR-rich organs, such as the pancreas. Furthermore, nephrotoxicity due to the tubular reabsorption of peptides as well as radiation-induced acute myelotoxicity need to be carefully evaluated. In this regard, a previous preclinical study demonstrated how the administration of fractionated doses of [^213^Bi]Bi-DOTA-PESIN resulted in lower renal toxicity and higher efficacy in a PC pre-clinical model (114). Moreover, a phase I dose escalation study with the compound [^212^Pb]Pb-DOTAM-GRPR1 is currently ongoing in patients with recurrent or metastatic GRPR-expressing tumors, including BC patients, and will contribute to elucidate such concerns by determining the single and multiple ascending doses of this novel GRPR-targeting radiopharmaceutical (NCT05283330) (105).

The results from other ongoing clinical trials are eagerly awaited, in particular the evaluation of the theranostic couple [^68^Ga]Ga/[^177^Lu]Lu-NeoBOMB1 in patients with GRPR-expressing malignancies, including ER-positive/HER2-negative breast cancer led by Novartis (86). Also, Clarity Pharmaceuticals will soon recruit patients for the assessment of the theranostic pair [^64^Cu]Cu-SAR-BBN/[^67^Cu]Cu-SAR-BBN for imaging and treatment of PC (NCT05633160) (115). Since the company has already concluded a clinical trial on the safety and diagnostic value of [^64^Cu]Cu-SAR-BBN PET/CT for ER/PR-positive metastatic BC, it is likely that the evaluation of the GRPR-targeting theranostic couple using copper radioisotopes will soon be extended also to BC patients (97).

## Author contributions

AD’O and EG contributed to conception and design of the review. AD’O organized and collected the data and wrote the first draft of the manuscript. All authors contributed to manuscript revision and editing and have approved the final submitted version.
